# Phase Synchronization of Hemodynamic Variables at Rest and after Deep Breathing Measured during the Course of Pregnancy

**DOI:** 10.1371/journal.pone.0060675

**Published:** 2013-04-05

**Authors:** Manfred Georg Moertl, Helmut Karl Lackner, Ilona Papousek, Andreas Roessler, Helmut Hinghofer-Szalkay, Uwe Lang, Vassiliki Kolovetsiou-Kreiner, Dietmar Schlembach

**Affiliations:** 1 Department of Obstetrics and Gynecology, Medical University of Graz, Graz, Austria; 2 Department of Obstetrics and Gynecology, Clinical Center, Klagenfurt, Austria; 3 Department of Physiology, Medical University of Graz, Graz, Austria; 4 Department of Medical Engineering, Graz University of Technology, Graz, Austria; 5 Department of Psychology, Biological Psychology Unit, Karl-Franzens University, Graz, Austria; 6 Department of Obstetrics and Gynecology, Friedrich Schiller University, University Clinics Jena, Jena, Germany; Max-Delbrück Center for Molecular Medicine (MDC), Germany

## Abstract

**Background:**

The autonomic nervous system plays a central role in the functioning of systems critical for the homeostasis maintenance. However, its role in the cardiovascular adaptation to pregnancy-related demands is poorly understood. We explored the maternal cardiovascular systems throughout pregnancy to quantify pregnancy-related autonomic nervous system adaptations.

**Methodology:**

Continuous monitoring of heart rate (R-R interval; derived from the 3-lead electrocardiography), blood pressure, and thoracic impedance was carried out in thirty-six women at six time-points throughout pregnancy. In order to quantify in addition to the longitudinal effects on baseline levels throughout gestation the immediate adaptive heart rate and blood pressure changes at each time point, a simple reflex test, deep breathing, was applied. Consequently, heart rate variability and blood pressure variability in the low (LF) and high (HF) frequency range, respiration and baroreceptor sensitivity were analyzed in resting conditions and after deep breathing. The adjustment of the rhythms of the R-R interval, blood pressure and respiration partitioned for the sympathetic and the parasympathetic branch of the autonomic nervous system were quantified by the phase synchronization index γ, which has been adopted from the analysis of weakly coupled chaotic oscillators.

**Results:**

Heart rate and LF/HF ratio increased throughout pregnancy and these effects were accompanied by a continuous loss of baroreceptor sensitivity. The increases in heart rate and LF/HF ratio levels were associated with an increasing decline in the ability to flexibly respond to additional demands (i.e., diminished adaptive responses to deep breathing). The phase synchronization index γ showed that the observed effects could be explained by a decreased coupling of respiration and the cardiovascular system (HF components of heart rate and blood pressure).

**Conclusions/Significance:**

The findings suggest that during the course of pregnancy the individual systems become increasingly independent to meet the increasing demands placed on the maternal cardiovascular and respiratory system.

## Introduction

During pregnancy the maternal cardiovascular system (CVS) undergoes profound changes important to assure a normal pregnancy outcome [1]–[4]. The capacity of cardiovascular regulation to operate effectively under varying conditions depends on the integrity of parasympathetic and sympathetic systems and neurohormonal mechanisms. However the role of the autonomic nervous system (ANS) in the cardiovascular adaptation to pregnancy-related demands is poorly understood [5]–[7].

The analysis of heart rate variability (HRV), blood pressure variability (BPV), and baroreflex sensitivity (BRS) has become a powerful tool for the assessment of autonomic control [8]–[10]. In the field of gynecology, these techniques are particularly suitable for pregnant women because virtually non-invasive devices allow studying the profound changes of maternal heart rate (R-R interval), blood pressure (BP) and respiration (RESP) during pregnancy [11]–[15]. Various mathematical methods have been used with the objective to appropriately describe the cardiovascular changes and their mechanisms during gestation [16]–[23].

It is widely accepted that respiratory activity modifies heart rate and blood pressure oscillations, and numerous recent studies demonstrated interactions among respiration, heart rate and blood pressure [24], [25]. In addition, blood pressure waves with a fundamental frequency of 0.1 Hz (Mayer waves) modulate the constant intrinsic rhythm of the cardiac pacemaker [26]. The extent of these fluctuations is situation and age dependent [27], [28].

Schaefer et al. (1998) showed that the weak interactions between the human heart and respiratory systems can be identified by the concept of phase synchronization of chaotic oscillators [29].For example, the nonlinear dynamics of cardiovascular ageing could be demonstrated using this concept [30]. Furthermore, synchronization of the main heart rhythm and the rhythm of slow regulation of blood pressure with respiration has been shown [31]. Recent studies demonstrated a decoupling of the CVS and the respiratory system under stress conditions such as exercise or mental stress [32], [33].

The purpose of this study was to examine the cardiovascular pregnancy adaptations by analyzing the adjustment of the rhythms of the R-R interval (RRI), blood pressure and respiration throughout gestation. We employed the concept of analytic signals to examine the phase synchronization, which we recently used to demonstrate how mental stress affects the functional interaction of autonomic nervous activity [32]. Phase synchronization is a fundamental nonlinear phenomenon that can be treated as an emergence of some relation between functionals of two processes due to interaction [34]. A change of synchronization reflects variation in the state of a complex system, like the CVS and therefore may provide important physiological information [29], [35].

We examined cardiovascular variables indicative of the sympathetic and parasympathetic branches of the ANS during rest and after deep breathing (DB), hypothesizing that phase synchronization, quantified by the synchronization index (γ), can be used to quantify pregnancy-related changes in the ANS. Surrogate data analysis was used to distinguish between causal relationships and those that occur by pure chance [36], [37].

## Materials and Methods

### Ethics Statement

The study was performed in accordance with the 1964 Declaration of Helsinki and was approved by the Ethics Committee of the Medical University of Graz. Written informed consent was obtained from all participants.

### Participants

Pregnant women who underwent first trimester screening were asked to participate in the study. Forty-two women gave written informed consent to participate in the study. Women with pre-existing diseases such as insulin-dependent diabetes or cardiovascular or renal diseases, and/or pregnancy related complications and disorders such as preeclampsia were excluded from the study. All women had singleton pregnancies, and consecutively had a normal pregnancy outcome.

### Experimental Procedure

After participants were familiarized with the test protocol, equipment and personnel, electrodes were attached and patients were positioned in the 15° left lateral position, ensuring a continuous venous blood flow to the heart. During the whole procedure the participants were asked not to talk or make abrupt movements. The study protocol consisted of a short adaptation period of 20 min, 10 min recording at rest and 1 min deep breathing (DB; 6 breaths/min) followed by another 5 min of rest. For analysis, five minutes epochs preceding DB and following DB were used.

Measurements were performed longitudinally throughout gestation at time intervals of 4–5 weeks. Measurement occasions were grouped into six categories according to gestational age. Linear interpolation was used to constitute equivalent time points at week 12 (range 11^+2^–14^+0^), 16 (15^+0^–17^+4^), 20 (18^+0^–22^+2^), 25 (23^+4^–27^+0^), 30 (28^+3^–32^+1^) and 35 (33^+0^–37^+0^) across participants.

### Data acquisition and Preprocessing

Continuous monitoring of BP (sampling rate, sr = 100 Hz, BP_range_ = 50–250 mmHg, ±5 mmHg), RRI (3-lead electrocardiography, sr = 1 kHz, f_cut-off_ = 0.08–150 Hz) and thoracic impedance were carried out with the Task Force® Monitor (TFM®; CNSystems, Medizintechnik AG, Graz, Austria) [38]. Continuous BP was derived from the finger using a refined version of the vascular unloading technique and corrected to absolute values with oscillometric BP measurement by the TFM® [38]. Electrodes were placed at the neck and thoracic regions, the latter specifically at the midclavicular line at the xiphoid process level.

To obtain RRI and BP time series with equidistant time steps, the beat-to-beat values were resampled at 4 Hz, using piecewise cubic spline interpolation after artifact correction. Single artifacts were replaced by interpolation and its appearance recorded. Furthermore, the respiratory signal was derived from the thoracic impedance and down sampled to 4 Hz to obtain corresponding sampling times as RRI and BP. Due to the strict artifact handling – only five minute epochs with at least 95% valid R-R interval (RRI) data were accepted – data of 36 out of 42 women were used.

Time domain indexes of heart rate variability (HRV) were computed as the standard deviation of normal-to-normal beat (SDNN) and root mean squared successive differences (rMSSD) of R-R intervals. Time domain indexes of blood pressure variability (BPV) were computed as the standard deviation (SD).

For frequency domain indexes of RRI and systolic and diastolic BP (SBP, DBP), we used Fast Fourier Transform with a Hanning window for spectral analysis of cardiovascular signals on the blocks of 5 min epochs, after resampling and removing the trend of 2^nd^ order. Low frequency (LF) was defined as 0.04–0.15 Hz, high frequency (HF) was defined as 0.15–0.40 Hz, according to published recommendations [39]. Because of skewed distributions of frequency domain indexes, a natural logarithmic transformation was applied to the LF-components of RRI (ln(LF_RRI_), SBP (ln(LF_SBP_)), and DBP (ln(LF_DBP_)), for the HF-component of RRI (ln(HF_RRI_)) and the LF/HF ratio (ln(LF/HF)_RRI_).

The sequence technique was used for the assessment of baroreceptor reflex sensitivity (BRS) [40]. Usually, sequences of three to six consecutive cardiac beats are sought in which an increase in SBP is accompanied by an increase in RRI, or in which a decrease in SBP is accompanied by a decrease in RRI. The regression line between the SBP and the RRI values produces an estimate of BRS. In our study an equivalent change in RRI and SBP for at least three consecutive cardiac cycles was defined as a regulatory event if the following criteria were fulfilled: (1) RRI variations >4 ms; (2) SBP changes >1 mmHg.

**Figure 1:**
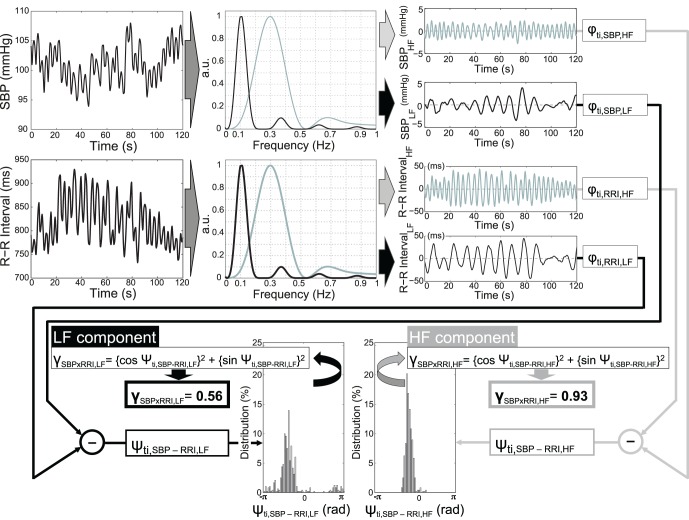
Steps to compute the phase synchronization: First, the signals, here systolic blood pressure (SBP) and R-R interval (RRI), were band-pass filtered. The black line denotes the filter characteristic of the band pass filter for the LF-component, the grey line denotes the filter characteristic of the band pass filter for the HF-component. The Hilbert transformation was employed to calculate the phase of the filtered signals. The rectangles with the symbol φ_ti_ denote the Hilbert transformation and the resulting phase at the time t_i_. In the final step the phase difference Ψ_ti_ was used to quantify the phase synchronization γ for the related period.

To obtain patterns containing only LF or HF components, time series were band-pass filtered. The filtering process must not alter the phase of the time series; therefore, we used a two-step approach. First, a moving average with two windows of different lengths was calculated (16 and 48 samples, i.e., 4 and 12 s for the LF component and 7 and 16 samples, i.e. 1.75 and 4 s for the HF component; see Figure 1 for filter characteristics). Then, we obtained the filtered time series by subtracting the longer window length moving average time series from the shorter window length.

### Analysis Procedure Using Phase Synchronization

The analysis of synchronization, e.g., of RRI and SBP is based upon the weak coupling of two chaotic systems. Each oscillator can be described by its amplitude and phase as a function of time. For the purpose of our study, only a phase (but not amplitude) needed to be defined for a time series that contains oscillations in a narrow frequency band. Therefore, for the analysis of phase relations we had to estimate phases from data. In recent studies, time series phase definition was done using the concept of analytic signals [29], [41]. We used the MATLAB-function HILBERT (MATLAB®, MathWorks Natick, Massachusetts, USA) to compute the so-called discrete-time analytic signal **X** with X = Xr+i*Xi such that **Xi** is the Hilbert transform of real vector **Xr**, which is in our case the band-pass filtered time series. To admit a clear physical interpretation, which is given only for narrow band signals, we used the band-pass filtered time series [35]. We calculated the phase of the resulting signal **X** at every time point with the MATLAB-function ANGLE.

In the next step, the difference between two given phase vectors for the interpolated bivariate data series, e.g., in this case between RRI and SBP, was calculated. The time series are defined as synchronized if this phase difference is constant over time. In case of synchronization, the distribution of the phase difference Ψ*(t_i_)* shows a definite maximum. The distribution of Ψ*(t_i_)* is quantified by the synchronization index γ defined by

where the brackets {…} denote an average and *t_i_* the sample times. Theoretically, if the synchronization index γ = 1, then both time series are completely synchronized in a statistical sense, while in the case of γ = 0 both time series are completely desynchronized, i.e., the values of Ψ*(t_i_)* are equally distributed in the range of [-π, π]. Phase synchronization thus provides a quantitative indicator of the coordinated behavior of pairs of systems (see Figure 1).

This approach was implemented to calculate the phase for continuous signal analysis partitioned in LF and HF during rest and post stress. Deep breathing, a sensitive non-invasive maneuver to quantify cardiac parasympathetic activity, was used to mediate cardiovascular reflex responses to standard stimuli [42].

### Analysis Procedure with Surrogate Data

For real-life data, the lower bound of γ has to be estimated because, even in the absence of any coordination, synchronized patterns may appear by chance. To accomplish this task, the method of surrogate data analysis for bivariate data has been employed [28]. Surrogate data analysis is a widely used approach in the field of nonlinear dynamics. The essence of surrogate analysis is the construction of a large (surrogate) data set derived from the original (real) data. This is typically achieved by randomizing a data feature, the influence of which is under investigation, while all other features of the data are preserved. The statistical observation of a difference in the measured data feature between the real and the surrogate data indicates that this difference is related to that specific feature which is absent in the surrogates. The surrogate data were created from the original signal by computing the Fourier Transform and randomizing the phase in the frequency domain by multiplying the complex values with e^iФ^, with Ф from the interval [0,2 π], independent from the frequency. Following this randomization in the frequency domain, the data were transformed back to the time domain by inverse Fourier Transformation. Such data have the same mean, standard deviation, and power spectrum as the original data. However the temporal structure is different from the original data.

### General Analysis Procedure

For each bivariate data analysis, from one original dataset, 100 datasets were prepared as described above and the 100 corresponding γ_signal1 x signal2,surrogate_ were calculated. The 95^th^ percentile of γ_signal1 x signal2,surrogate_ was used as so-called “surrogate” data for the statistical analysis. To determine if there is a difference between cardiovascular synchronization measures calculated with original and surrogate-data, analyses of variance were conducted for the resting condition, with *“week”* (week 12, week 16, week 20, week 25, week 30, week 35, within-subjects factor) and *“type of data”* (original data, surrogate data approach; within-subjects factor) as independent variables and the cardiovascular synchronization measures as the dependent variables.

To evaluate the effects of pregnancy on cardiovascular responses, multivariate analyses of variance (ANOVAs) for repeated measurements were conducted, with *“week”* and *“condition”* (rest, post stress; within-subjects factor) as independent variables, and the cardiovascular synchronization measures as the dependent variables. Separate analyses were conducted for variables of heart rate variability (HRV), variables of blood pressure variability (BPV), variables related to thoracic impedance and BRS as well as for the LF- and HF- component of the cardiovascular synchronization measures, where the cardiovascular synchronization measures were different from the surrogate data approach.

Potential influences of minor irregularities in the distribution of scores on the statistical results are ruled out by the effect of the Central Limit Theorem [43]. Inspection of the distributions ensured that none of the analyzed variables showed strongly deviating scores.

## Results

Data presented here are from 36 pregnant women of age 31±5 years (mean ± SD; range: 19–39 years), weight 62±9 kg (47–85 kg), height 166.5±5.5 cm (152–180 cm), and body mass index (BMI) 22.3±3.4 kg/m^2^ (18.1–30.1 kg/m^2^). Body weight at week 12 was 63±9 kg (50–89 kg) and increased to 74±10 kg (60–102 kg at week 35).

### Cardiovascular and Hemodynamic Variables

The multivariate analysis of HRV variables revealed significant changes for the main effects of *“week”* (*F*(25,11) = 5.2, *p*<.01) and “condition” (rest, post DB; *F*(5,31) = 11.3, *p*<. 001), as well as a significant interaction “week by condition” (*F*(25,11) = 3.5, *p*<.05), i.e. the effect of DB on HRV variables varied with the week of gestation.

The heart rate (HR) and ln(LF/HF)_RRI_ increased to 35 weeks’ gestation, indicated by a linear trend (*F*(1,35) = 48.1, *p*<.001, *η*_p_^2^ = 0.58, *F*(1,35) = 36.8, *p*<.001, *η*_p_^2^ = .51, respectively), whereas SDNN (F(1,35) = 13.3, *p*<.01, *η*_p_^2^ = .28), rMSSD (F(1,35) = 17.9, *p*<.001, *η*_p_^2^ = .34), ln(LF_RRI_) (*F*(1,35) = 22.2, *p*<.001, *η*_p_^2^ = .39) and ln(HF_RRI_) (*F*(1,35) = 55.4, *p*<.001, *η*_p_^2^ = .61) decreased with advancing gestational age. With advancing gestational age the effects of DB, indicated by “week by condition” interaction, was significantly different for HR (*F*(5,175) = 2.7, *p*<.05, *η*_p_^2^ = .07), rMSSD (*F*(5,175) = 2.4, *p*<.05, *η*_p_^2^ = .06), and ln(LF/HF)_RRI_ (*F*(5,175) = 2.5, *p*<.01, *η*_p_^2^ = .07). Means ± SD as well as the subsequently performed univariate *F*-tests are reported in Table 1.

**Table 1:** Heart rate and heart rate variability variables (mean ± SD) of participants and statistical results.

	week 12	week 16	week 20	week 25	week 30	week 35		*ANOVA*		
***HR (bpm)***									*p*	*η_p_^2^*
*rest*	76.1±9.3	77.9±8.3	81.2±8.1	83.7±8.8	86.1±10.0	86.1±10.5	*week*	*F*_(5,175)_ = 22.7	*<.001*	.39
*post DB*	75.6±9.4	78.5±8.4	81.3±8.3	82.2±7.9 *	85.0±10.0	84.7±9.9	*condition*	*F*_(1,35)_ = 5.9	*<.05*	.14
							*week x condition*	*F*_(5,175)_ = 2.7	*<.05*	.07
***SDNN (ms)***										
*rest*	47.6±20.2	42.1±16.8	42.1±17.9	39.9±13.4	36.7±19.5	36.8±19.2	*week*	*F*_(5,175)_ = 6.9	*<.001*	.16
*post DB*	50.6±20.4	47.9±20.5	45.5±19.4	39.1±13.8	40.5±20.5	40.0±15.9	*condition*	*F*_(1,35)_ = 22.4	*<.001*	.39
							*week x condition*	*F*_(5,175)_ = 0.5	* = .80*	.01
***rMSSD (ms)***										
*rest*	37.5±23.4	31.7±17.3	27.2±16.6	20.9±9.9	21.7±21.5	19.8±14.0	*week*	*F*_(5,175)_ = 10.0	*<.001*	.22
*post DB*	36.0±23.4	30.9±18.8	27.1±17.6	22.9±11.4 *	22.3±22.4	22.0±14.6	*condition*	*F*_(1,35)_ = 0.7	* = .42*	.02
							*week x condition*	*F*_(5,175)_ = 2.4	*<.05*	.06
***ln(LF_RRI_) (ms^2^)***										
*rest*	6.0±1.1	5.9±0.9	6.0±0.9	5.6±0.7	5.6±0.9	5.5±0.9	*week*	*F*_(5,175)_ = 7.8	*<.001*	.18
*post DB*	6.3±1.0	6.2±0.9	6.1±0.9	5.8±0.7	5.8±0.9	5.7±0.9	*condition*	*F*_(1,35)_ = 17.3	*<.001*	.33
							*week x condition*	*F*_(5,175)_ = 0.3	* = .90*	.01
***ln(HF_RRI_) (ms^2^)***										
*rest*	5.8±1.3	5.6±1.1	5.3±1.0	5.0±0.9	4.7±1.2	4.7±1.1	*week*	*F*_(5,175)_ = 19.4	*<.001*	.36
*post DB*	5.8±1.1	5.5±1.1	5.3±0.9	5.0±1.0	4.8±1.2	4.9±1.1	*condition*	*F*_(1,35)_ = 0.3	* = .59*	.01
							*week x condition*	*F*_(5,175)_ = 1.6	* = .16*	.04
***ln(LF/HF)_RRI_ (-)***										
*rest*	0.2±0.8	0.3±0.6	0.7±0.6	0.6±0.6	0.9±0.6	0.9±0.6	*week*	*F*_(5,175)_ = 11.5	*<.001*	.25
*post DB*	0.5±0.6 *	0.7±0.5 *	0.8±0.5	0.8±0.7	1.0±0.6	0.9±0.6	*condition*	*F*_(1,35)_ = 14.6	*<.01*	.29
							*week x condition*	*F*_(5,175)_ = 2.5	*<.05*	.07

HR = heart rate; SDNN = standard deviation of normal-to-normal beat; rMSSD = root mean squared successive differences of R-R intervals; LF = low frequency (0.04–0.15 Hz); HF = low frequency (0.15–0.40 Hz); ln = natural logarithmic transformation; DB = deep breathing. *denotes a significant difference (*p<.05) between rest and post DB* in case of a significant univariate interaction effect.

**Figure 2:**
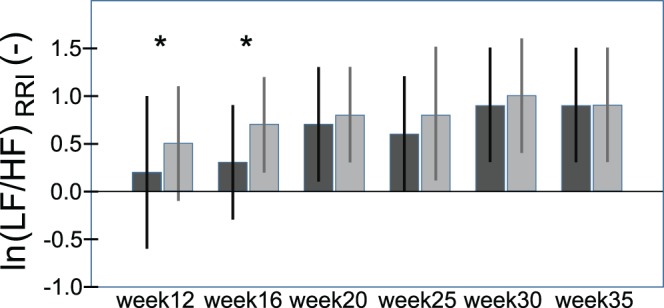
LF/HF ratio (ln(LF/HR)_RRI_; mean ± SD) throughout gestation: Black bars depict the values of the variables in rest, the grey bars show the post stress (deep breathing) condition (RRI = R-R interval; LF = low frequency; HF = high frequency; ln = natural logarithmic transformation). *denotes a significant difference (*p<.05) between rest and post DB* in case of a significant univariate interaction effect.

The LF/HF ratio as a measure of characterizing the autonomic state resulting from sympathetic and parasympathetic influences increases with advancing gestational age, whereby the effect of DB is decreasing throughout gestation, i.e. the differences between HRV parameters before and after DB found at lower gestational age diminish with advancing pregnancy (see Figure 2).

By multivariate analysis of BPV significant changes for the main effects of “week” (*F*(30,6) = 4.3, *p*<.05) and “condition” (rest, post DB; *F*(6,30) = 10.0, *p*<.001) but not for the “week by condition” interaction (*F*(30,6) = 0.9, *p* = .60) were detectable. SBP and DBP were significantly higher in week 35 compared to week 16 and week 20 indicated by a linear trend (*F*(1,35) = 15.9, *p*<.001, *η*_p_^2^ = .31, *F*(1,35) = 14.9, *p*<.001, *η*_p_^2^ = .30) and also by a quadratic trend (*F*(1,35) = 8.5, *p*<.01, *η*_p_^2^ = .20, *F*(1,35) = 8.1, *p*<.01, *η*_p_^2^ = .19). Means ± SD as well as the subsequently performed univariate *F*-tests are reported in Table 2.

**Table 2:** Blood pressure and blood pressure variability variables (mean ± SD) of participants and statistical results.

	week 12	week 16	week 20	week 25	week 30	week 35		*ANOVA*		
***SBP (mm Hg)***									*p*	*η_p_^2^*
*rest*	105.1±11.4	100.0±12.7	101.7±14.8	103.2±14.9	106.3±12.6	108.0±11.6	*week*	*F*_(5,175)_ = 5.0	*<.001*	.13
*post DB*	105.6±9.2	103.5±12.5	103.5±13.1	105.2±13.9	109.1±10.8	110.1±11.1	*condition*	*F*_(1,35)_ = 13.8	*<.01*	.28
***SD_SBP_ (mm Hg)***										
*rest*	3.9±1.8	3.8±1.0	4.6±1.6	4.3±1.6	5.0±2.1	4.8±1.6	*week*	*F*_(5,175)_ = 5.8	*<.001*	.14
*post DB*	4.3±1.6	4.7±1.6	5.7±2.2	5.0±1.6	5.1±1.5	5.5±1.9	*condition*	*F*_(1,35)_ = 24.8	*<.001*	.42
***ln(LF_SBP_) (mm Hg^2^)***										
*rest*	0.7±0.7	0.8±0.6	1.0±0.7	0.9±0.7	1.1±0.7	1.2±0.6	*week*	*F*_(5,175)_ = 6.5	*<.001*	.16
*post DB*	0.7±0.6	1.0±0.6	1.2±0.6	1.0±0.6	1.3±0.7	1.3±0.8	*condition*	*F*_(1,35)_ = 7.4	*<.05*	.18
***DBP (mm Hg)***										
*rest*	63.3±10.3	60.2±10.5	61.0±11.7	62.8±12.0	65.1±10.1	67.0±11.8	*week*	*F*_(5,175)_ = 5.8	*<.001*	.14
*post DB*	63.8±7.6	62.4±10.1	62.1±10.9	64.4±10.2	67.0±8.7	68.9±10.7	*condition*	*F*_(1,35)_ = 12.1	*<.01*	.26
***SD_DBP_ (mm Hg)***										
*rest*	3.5±1.4	3.1±0.9	3.6±1.2	3.4±1.2	3.6±1.7	3.4±1.0	*week*	*F*_(5,175)_ = 2.1	* = .07*	.06
*post DB*	3.6±1.0	4.3±1.3	4.7±1.4	4.2±1.4	4.1±1.3	4.2±1.5	*condition*	*F*_(1,35)_ = 48.8	*<.001*	.58
***ln(LF_DBP_) (mm Hg^2^)***										
*rest*	0.7±0.8	0.8±0.6	0.8±0.7	0.7±0.6	0.8±0.6	0.7±0.7	*week*	*F*_(5,175)_ = 16	* = .17*	.04
*post DB*	0.9±0.5	1.0±0.6	1.1±0.6	0.8±0.6	1.0±0.5	0.8±0.7	*condition*	*F*_(1,35)_ = 11.8	*<.01*	.25

SBP = systolic blood pressure; DBP = diastolic blood pressure; SD = standard deviation; LF = low frequency (0.04–0.15 Hz); ln = natural logarithmic transformation; DB = deep breathing. Note : Multivariate analyses of variance significant for main effects only.

Thoracic impedance was significantly influenced by “week” (*F*(15,21) = 5.9, *p*<.001) and “condition” (F(3,33) = 34.4, *p*<.001) but not by the “week by condition” interaction (*F*(15,21) = 2.0, *p* = . 07). The respiratory related change of the thoracic impedance, indicating the tidal volume, increased during the course of pregnancy denoted by a linear trend (*F*(1,35) = 55.2, *p*<.001, *η*_p_^2^ = .61), whereas no difference in the respiratory frequency was observed. The BRS decreased during the course of pregnancy indicated by a linear (*F*(1,35) = 35.7, *p*<.001, *η*_p_^2^ = .51) and quadratic trend (*F*(1,35) = 4.5, *p*<.05, *η*_p_^2^ = .11), however no influence of DB was observed. In addition, no differences in the effect of DB were seen for the reported variables throughout gestation (Table 3).

**Table 3:** Thoracic impedance variables and baroreflex sensitivity (mean ± SD) of participants and statistical results.

	week 12	week 16	week 20	week 25	week 30	week 35		*ANOVA*		
***Thoracic impedance (Ohm)***									*p*	*η_p_^2^*
*rest*	35.8±5.5	35.9±5.2	36.3±4.7	37.5±5.2	36.5±3.4	34.9±3.8	*week*	*F*_(5,175)_ = 2.9	*<.05*	.08
*post DB*	35.8±5.7	35.6±5.1	36.2±4.7	37.3±5.3	36.3±3.2	34.6±3.7	*condition*	*F*_(1,35)_ = 6.7	*<.05*	.16
***Delta Z_ 0,RESP_ (Ohm)***										
*rest*	0.43±0.12	0.44±0.16	0.48±0.15	0.52±0.13	0.58±0.16	0.63±0.18	*week*	*F*_(5,175)_ = 26.7	*<.001*	.43
*post DB*	0.43±0.14	0.43±0.16	0.48±0.16	0.51±0.15	0.56±0.15	0.58±0.16	*condition*	*F*_(1,35)_ = 3.1	* = .09*	.08
***RF (1/min)***										
*rest*	18.8±2.7	18.5±2.5	18.7±2.6	18.6±2.2	17.9±2.4	18.3±2.0	*week*	*F*_(5,175)_ = 1.2	* = .32*	.03
*post DB*	17.2±2.5	16.7±2.1	17.1±2.6	17.4±2.3	16.9±2.1	17.3±2.0	*condition*	*F*_(1,35)_ = 87.0	*<.001*	.71
***BRS (ms/mm Hg)***										
*rest*	18.4±9.0	16.6±7.9	14.3±6.6	11.9±5.2	11.1±8.8	10.1±5.4	*week*	*F*_(5,175)_ = 16	*<.001*	.61
*post DB*	18.2±10.1	15.9±7.6	13.7±7.2	11.8±5.9	11.0±9.9	10.5±5.9	*condition*	*F*_(1,35)_ = 11.8	* = .63*	.01

Delta Z_0,_RESP = change of thoracic impedance (Z0) driven by respiration; RF = respiratory frequency; BRS = baroreflex sensitivity; DB = deep breathing. Note : Multivariate analyses of variance significant for main effects only.

### Surrogate Data and the Analytic Signals Analysis

#### LF-components

A significant effect for “type of data” for the phase synchronization of SBP and RRI, DBP and RRI as well as SBP and DBP was detectable. Phase synchronization between SBP and RESP was higher for surrogate data compared to real data. Furthermore, all phase synchronization indices for signals related to the LF component of RESP were on average less than 0.1, indicating a non-significant effect. Therefore, no further analysis was carried out with the LF-components of the synchronization indices γ_RESPxRRI,LF_, γ_RESPxSBP,LF_ and γ_RESPxDBP,LF_.

Multivariate analysis of the LF-components of the synchronization variables γ_SBPxRRI,LF_, γ_DBPxRRI,LF_ and γ_SBPxDBP,LF_ revealed a significant main effect of “week” (*F*(15,21) = 2.7, *p*<.05, *η*_p_^2^ = .66), but not of “condition” (*F*(3,33) = 2.7, *p* = .06, *η*_p_^2^ = .20). The subsequently performed univariate *F*-tests revealed that the main effect of *“week”* held for γ_SBPxRRI_ and γ_DBPxRRI_. Scores decreased for γ_SBPxRRI,LF_, (linear trend, *F*(1,35) = 10.7, *p*<.01, *η*_p_^2^ = .24; quadratic trend, *F*(1,35) = 5.3, *p*<.05, *η*_p_^2^ = .13) and γ_DBPxRRI,LF_ (linear trend, *F*(1,35) = 11.6, *p*<.01, *η*_p_^2^ = .25), whereas γ_SBPxDBP,LF_ remained remarkably stable during the course of pregnancy (Table 4).

**Table 4:** Phase synchronization indices of the *LF-components* (mean ± SD) of participants and statistical results.

LF components										
	week 12	week 16	week 20	week 25	week 30	week 35		ANOVA		
**γ**_SBPxRRI,LF_									p	η_p_^2^
rest	0.34±0.13	0.30±0.10	0.28±0.11	0.27±0.12	0.29±0.12	0.28±0.12	week	F_(5,175)_ = 5.0	<.001	.13
post DB	0.31±0.13	0.31±0.12	0.27±0.10	0.25±0.12	0.26±0.12	0.25±0.13	condition	F_(1,35)_ = 3.4	= .07	.09
**γ**_DBPxRRI,LF_										
rest	0.42±0.13	0.39±0.13	0.37±0.14	0.36±0.13	0.37±0.14	0.34±0.14	week	F_(5,175)_ = 5.0	<.001	.12
post DB	0.38±0.13	0.37±0.12	0.34±0.12	0.31±0.12	0.34±0.12	0.31±0.12	condition	F_(1,35)_ = 7.9	<.01	.18
**γ**_SBPxDBP,LF_										
rest	0.66±0.15	0.67±0.12	0.63±0.12	0.63±0.13	0.65±0.12	0.63±0.15	week	F_(5,175)_ = 1.2	= .33	.03
post DB	0.62±0.17	0.65±0.14	0.62±0.13	0.61±0.13	0.63±0.12	0.66±0.14	condition	F_(1,35)_ = 2.3	= .14	.06

LF = low frequency; γ = synchronization index; RRI = R-R interval; SBP = systolic blood pressure; DBP = diastolic blood pressure; DB = deep breathing. Note : Multivariate analyses of variance significant for main effect “week” only.

#### HF-components

A significant effect for “type of data” for the phase synchronization variables at HF was observed. Significantly higher synchronization was seen for real data signals than for surrogate data signals.

Multivariate analysis of the synchronization variables γ_SBPxRRI,HF_, γ_DBPxRRI,HF_ and γ_SBPxDBP,HF_ of HF-components revealed significant effects of “week” (*F*(15,21) = 10.1, *p*<.001, *η*_p_^2^ = .88) and “condition” (*F*(3,33) = 26.7, *p*<.001, *η*_p_^2^ = .70) but not “week by condition” interaction (*F*(15,21) = 1.3, *p* = .27, *η*_p_^2^ = .49). γ_SBPxRRI,HF_ and γ_DBPxRRI,HF_ decreased to 35 weeks’ gestation, indicated by a linear trend (*F*(1,35) = 19.9, *p*<.001, *η*_p_^2^ = 0.36 and *F*(1,35) = 12.4, *p*<.01, *η*_p_^2^ = .26, respectively) and quadratic trend (*F*(1,35) = 11.2, *p*<.01, *η*_p_^2^ = 0.24, *F*(1,35) = 4.2, *p*<.05, *η*_p_^2^ = .11), whereas γ_SBPxDBP,HF_ increased (linear trend, *F*(1,35) = 40.6, *p*<.001, *η*_p_^2^ = .54). Furthermore, these variables were lower after DB compared to the resting condition before.

For the HF-components related to RESP (the synchronization indices γ_RESPxRRI,HF_, γ_RESPxSBP,HF_ and γ_RESPxDBP,HF_) the multivariate analyses revealed significant effects of “week” (*F*(15,21) = 4.5, *p*<.01, *η*_p_^2^ = .76) and “condition” (*F*(3,33) = 26.0, *p*<.001, *η*_p_^2^ = .70), as well as differences of the DB effect “week by condition” interaction) in resting and post stress condition (*F*(15,21) = 2.3, *p*<.05, *η*_p_^2^ = .63).

**Figure 3:**
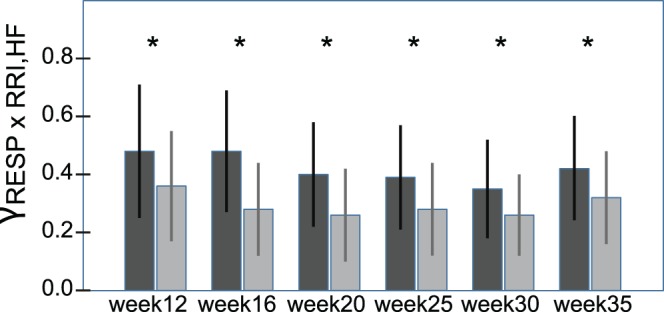
Time course of the phase synchronization index γ of R-R interval and respiration(HF-components; mean ± SD) throughout gestation: Black bars (mean ± SD) depict the values of the variables in rest, grey bars (mean ± SD) show the post stress (deep breathing) condition. (γ = synchronization index; RRI = R-R interval; RESP = respiration; HF = high frequency). *denotes a significant difference (*p<.05) between rest and post DB* in case of a significant univariate interaction effect.

A significant decrease was observed in γ_RESPxRRI,HF_ (linear trend *F*(1,35) = 4.9, p<.05, *η*_p_^2^ = .12; quadratic trend *F*(1,35) = 10.9, *p*<.01, *η*_p_^2^ = .24), whereas γ_RESPxSBP,HF_ reached its nadir at mid-pregnancy, indicated by a quadratic trend only (*F*(1,35) = 16.6, *p*<.001, *η*_p_^2^ = .32). γ_RESPxDBP,HF_ increased during the course of pregnancy (linear trend *F*(1,35) = 8.3, *p*<.01, *η*_p_^2^ = .19; quadratic trend *F*(1,35) = 9.0, *p*<.01, *η*_p_^2^ = .21). Additionally, the synchronization indices γ_RESPxRRI,HF_, γ_RESPxSBP,HF_ and γ_RESPxDBP,HF_ were lower after DB compared to the resting condition before and the time course of γ_RESPxRRI,HF_ (see Figure 3), indicated by a significant interaction “week by condition”, (*F*(5,175) = 3., *p*<.01, *η*_p_^2^ = .08), were different, too (Table 5).

**Table 5:** Phase synchronization indices of the *HF-components* (mean ± SD) of participants and statistical results.

HF components										
	week 12	week 16	week 20	week 25	week 30	week 35		ANOVA		
***γ*** *_SBPxRRI,HF_*									p	η_p_^2^
*rest*	0.50±0.17	0.46±0.18	0.40±0.16	0.39±0.15	0.36±0.16	0.38±0.16	week	F_(5,175)_ = 9.0	<.001	.21
*post DB*	0.38±0.15	0.33±0.14	0.28±0.14	0.28±0.12	0.27±0.13	0.31±0.13	condition	F_(1,35)_ = 79.6	<.001	.69
***γ*** *_DBPxRRI,HF_*										
*rest*	0.48±0.19	0.39±0.19	0.41±0.17	0.45±0.17	0.48±0.16	0.50±0.17	week	F_(5,175)_ = 5.5	<.001	.14
*post DB*	0.41±0.17	0.33±0.15	0.34±0.14	0.37±0.15	0.42±0.14	0.43±0.15	condition	F_(1,35)_ = 67.4	<.001	.66
***γ*** *_SBPxDBP,HF_*										
*rest*	0.38±0.17	039±0.19	0.41±0.17	0.45±0.17	0.48±0.16	0.50±0.17	week	F_(5,175)_ = 9.9	<.001	.22
*post DB*	0.31±0.11	0.33±0.15	0.34±0.14	0.37±0.15	0.42±0.14	0.43±0.15	condition	F_(1,35)_ = 31.9	<.001	.48
***γ*** *_RESPxRRI,HF_*										
*rest*	0.48±0.23	0.48±0.21	0.40±0.18	0.39±0.18	0.35±0.17	0.42±0.18	week	F_(5,175)_ = 4.8	<.001	.12
*post DB*	0.36±0.19*	0.28±0.16*	0.26±0.16*	0.28±0.16*	0.26±0.14*	0.32±0.16*	condition	F_(1,35)_ = 73.5	<.001	.68
							week x condition	F_(5,175)_ = 3.2	<.01	.08
***γ*** *_RESPxSBP,HF_*										
*rest*	0.54±0.21	0.48±0.22	0.44±0.22	0.45±0.22	0.43±0.21	0.52±0.16	week	F_(5,175)_ = 4.3	<.01	.13
*post DB*	0.41±0.19	0.33±0.17	0.30±0.17	0.34±0.15	0.35±0.17	0.39±0.15	condition	F_(1,35)_ = 74.8	<.001	.68
							week x condition	F_(5,175)_ = 0.9	= .50	.02
***γ*** *_RESPxDBP,HF_*										
	0.34±0.18	0.34±0.20	0.30±0.20	0.35±0.18	0.36±0.18	0.43±0.19	week	F_(5,175)_ = 4.8	<.001	.13
	0.24±0.14	0.20±0.14	0.19±0.13	0.24±0.12	0.28±0.15	0.30±0.14	condition	F_(1,35)_ = 63.6	<.001	.65
							week x condition	F_(5,175)_ = 1.4	= .22	.04

LF = low frequency; γ = synchronization index; RRI = R-R interval; SBP = systolic blood pressure; DBP = diastolic blood pressure; RESP = respiration; DB = deep breathing. *denotes a significant difference (*p<.05) between rest and post DB* in case of significant a univariate interaction effect. Note : Multivariate analyses of variance of γ*_SBPxRRI,HF_*, γ*_DBPxRRI,HF_* and γ*_SBPxDBP,HF_* significant for main effects only.

15 women had no history of previous gestations, 12 women were coursing the second gestation, and 9 women had history of more than two gestations. The repetition of the analysis with “history of gestation” (no previous gestation vs. previous gestations; between-subjects factor) as additional independent variable showed no additional results.

## Discussion

In the present study we confirmed the effects of pregnancy on physiological (i.e. cardiovascular and autonomous system related) measures [44], [45]. HR and LF/HF ratio increased throughout gestation and these effects were accompanied by a continuous loss of BRS [11]. The increases in heart rate and LF/HF ratio were associated with an increasing decline in the ability to flexibly respond to additional demands (i.e., diminished adaptive responses to deep breathing). The major finding using the phase synchronization index γ was that the observed effects could be explained by a decreased coupling of respiration and the cardiovascular system. Such desynchronization is known to occur under stress conditions [32]. Pregnancy is a cardiovascular stressor per se, therefore it seems likely that due to increasing demands during the course of pregnancy the individual systems become more independent to maintain proper function.

### Cardiovascular and Hemodynamic Variables

Our analysis suggests that the increase of HR throughout normal pregnancy is mainly driven by decreased parasympathetic activity, which confirms earlier observations [46]. Ekholm et al. found decreased parasympathetic responsiveness in early and mid-pregnancy with some restoration in the third trimester, along with diminished HRV, suggesting decreased parasympathetic and/or increased sympathetic nervous system tone [44], [47]. In our participants the decline of the sympathetic branch was lower than the decline of the parasympathetic branch, resulting in an increased LF/HF ratio. In addition, the time courses of the HRV variables at resting conditions preceding and following DB were different. DB is a sensitive non-invasive maneuver to quantify cardiac parasympathetic reactivity [42]. A challenge with DB (with 6 breaths/min) shifts the influence of the respiration to the LF (i.e. sympathetic) components. Therefore, it is likely, that the observed effect on the LF component after DB is due to the reactivation of the complex control loops after DB to reconstitute the original physiological status. Furthermore, at the end of pregnancy effects of DB on the LF/HF ratio were no more present, suggesting that the physiological ground status under resting conditions has already reached a high level because of the increased demands during pregnancy as such, resulting in only limited possibilities to respond to additional demands.

The results of our study confirm earlier observations that BP decreases until mid-pregnancy [1], [48], [49], probably due to an increased blood volume accompanied with decreased blood viscosity and consecutive vasodilatation [50], before it returns to or exceeds pre-pregnancy levels. Furthermore, the increase in systolic BPV confirms data of Blake et al. [17], again suggesting that mean arterial BP is the primary regulated variable during stress [51], [52].

The effect of breathing rate on the relationship between RRI and systolic pressure variability is a frequency-dependent phenomenon [53], [54]. However, in agreement to earlier observations [17], [46], respiratory frequency remained unaltered throughout pregnancy, apart from increased tidal volume leading to an increased minute volume. The analysis of BRS using the sequence method provides an index of autonomic nervous activity on RRI. There is evidence that the cardiac branch of the baroreflex that relates BP to RRI is one relevant source of parasympathetic influences and cardiac autonomic regulation [55], [56]. It is well-established that the BRS is diminished in essential hypertension and that this decrease precedes the onset of the disease [57], [58]. We also found a pronounced decrease of BRS throughout normal pregnancy which might be related to a reduction of vagal tone, rather an increase in sympathetic activity. These findings correspond to previous results of Blake et al. in normotensive pregnancy [17].

We used the non-invasive sequence technique to study the baroreceptor cardiac reflex, because this method identifies spontaneous cardiac sequences in which the baroreflex operates. However, not all the progressive changes in SBP are followed by reflex RRI modulation. Since physiological data are mostly non-stationary, the application of traditional techniques such as cross-spectrum and cross-correlation analysis or nonlinear statistical measures like mutual information do have its limitations. With the method used in this study, the analytic signal approach based on the Hilbert transform, it is possible to obtain unambiguously the phase difference for arbitrary signals [59].

### Surrogate Data and the Analytic Signals Analysis

#### LF-components

Respiration in our pregnant women was not synchronized with either RRI or BP. Although the heart rhythm and the rhythm of slow regulation of blood pressure can be synchronized with respiration [31], this was expected, because our participants were allowed to breath freely (0.2–0.4 Hz), and this is in agreement with earlier observations [60]. However, in addition it should be pointed out that in contrast to BRS, the analysis of the phase synchronization index indicates that the coupling of RRI and blood pressure slightly decreased after the stress, which might be related to the preceding respiration maneuver. Furthermore, the synchronization between RRI and BP decreased during the course of pregnancy supporting the results of BRS.

#### HF-components

A remarkable degree of coordination between SBP and RESP as well as RRI and RESP was observed during rest at first trimester (week 12). The coordination between RRI and SBP was also strong, suggesting that the coordination of RRI and SBP could be respiration driven. The synchronization of RRI and systolic as well as diastolic BP decreased with advancing gestational age, whereas the synchronization between SBP and DBP increased, supporting a declining influence of respiration on the coordination of RRI and BP.

Porta et al. reported, in nonpregnant patients, the coupling between RRI and SBP to gradually increase as a function of the tilt table inclination during the gradual sympathetic activation induced by a head up tilt in presence of a continual decrease of baroreflex sensitivity [61]. Furthermore, in nonpregnant patients, it has been reported that the coupling between RRI and RESP remained stable during head-up tilt [62]. The differences to our results may be explained by the challenges placed on the cardiovascular and autonomous system by pregnancy itself. Furthermore, using HRV variables Kuo et al. reported that the autonomic nervous activity changed towards a higher sympathetic and lower parasympathetic modulation as gestational age increased, which might be explained by the reduced influence of respiration on the coordination of RRI and BP observed in the present study [11].

Malberg et al. showed that taking into account a larger range of cardiovascular variables improves the prediction of pre-eclampsia [18]. Furthermore, it was reported that the respiratory influence on the heart rate and DBP was different between healthy subjects and PE patients [20]. Therefore, it seems likely that the respiration plays an important role in these processes [20].

The results of our study using the phase synchronization index in healthy women are in accordance with these observations. As mentioned above, the respiratory frequency remained unaltered during the course of pregnancy. However, the most pronounced effect of respiration on RRI and BP was observed at mid-pregnancy, which may be relevant to the detection of dynamical diseases such as pre-eclampsia that begins to occur at about week 20 of gestation. The present findings may also correspond to reports of increasing complexity of cardiac regulation mechanisms from mid-pregnancy onwards [63]. Nevertheless, respiratory frequency does not seem to be the only cause for changes in RRI variability as well as in systolic and diastolic BP fluctuations. Cardiovascular fluctuations might indicate mainly baroreflex-triggered changes in RRI [64], but respiratory sinus arrhythmia also might be due to a central mechanism or humoral phenomenon acting independently of hemodynamic changes [65]. These results support earlier observations of decreased parasympathetic responsiveness at mid-pregnancy with some restoration in the third trimester [44], [47]. Furthermore, the synchronization between RRI, BP and RESP was lower after DB compared to the resting condition preceding it. The observation, that the extent of the decline was less in later than in earlier phases of pregnancy, again supports that pregnancy is a cardiovascular stressor per se.

A limitation of the applied mathematical method may be that, although sympathetic and parasympathetic activity modulates the heart rate in different frequency bands, the LF components do not exclusively reflect sympathetic but to some extent also parasympathetic modulation. However, the sympathetic modulation should clearly outweigh the parasympathetic modulation [66]. Furthermore, Kreuz et al., comparing different approaches measuring synchronization in coupled model systems, concluded that it is difficult to a priori select the most suitable synchronization measure, because the underlying dynamics are usually not completely known [67]. However, although in the present study the used synchronization measure was not compared to other indexes, the empirical findings clearly indicate that the phase synchronization index γ is a valid and informative method of analysis for the applied purpose.

### Conclusion

Cardiovascular regulation has to maintain stable BP conditions in spite of higher blood volume, less viscosity and a huge arteriovenous shunt coming from the uteroplacental circulation, while respiratory regulation has to assure chemical homeostasis allowing for increased metabolic needs of the fetus, placenta and several maternal organs. In normal physiologic conditions the cardiovascular system is closely linked to the respiratory system. However, in stressful conditions such as exercise or mental stress a decoupling of both systems can be observed [32], [33], due to the complex control loops and the adaptation to changing demands. In the case of exercise the cardiovascular system is regulated to fulfill an adequate blood flow to the working muscles but only in the second place to maintain a stable blood pressure. This observation holds also for the control of breathing, which during exercise is not mainly triggered by the blood CO_2_ content, but by factors such as homeostasis of temperature or pH [68]. Pregnancy is a cardiovascular stressor per se, therefore it seems likely that the weakly coupled systems, due to increasing demands during the course of pregnancy, become less coordinated as they continue to function under these increasing demands. From a physiological point of view such uncoupling mechanisms might therefore be reasonable. Further studies should be done to analyze how coupling of these processes reoccurs post partum.
